# The GSK3β inhibitor BIS I reverts YAP-dependent EMT signature in PDAC cell lines by decreasing SMADs expression level

**DOI:** 10.18632/oncotarget.8437

**Published:** 2016-03-28

**Authors:** Natthakan Thongon, Ilaria Castiglioni, Chiara Zucal, Elisa Latorre, Vito D'Agostino, Inga Bauer, Michael Pancher, Alberto Ballestrero, Georg Feldmann, Alessio Nencioni, Alessandro Provenzani

**Affiliations:** ^1^ Laboratory of Genomic Screening, Centre for Integrative Biology, University of Trento, Trento, Italy; ^2^ Laboratory of Gene Expression and Muscular Dystrophy, San Raffaele Scientific Institute, Milan, Italy; ^3^ Department of Internal Medicine, University of Genoa, Genoa, Italy; ^4^ High Throughput Screening Facility, Centre for Integrative Biology, University of Trento, Trento, Italy; ^5^ Laboratory of Pancreatic Cancer Translational Research, Clinic University of Bonn, Bonn, Germany

**Keywords:** YAP, EMT, CTGF, PDAC, bisindolylmaleimides

## Abstract

The Yes-associated protein, YAP, is a transcriptional co-activator, mediating the Epithelial to Mesenchymal Transition program in pancreatic ductal adenocarcinoma (PDAC). With the aim to identify compounds that can specifically modulate YAP functionality in PDAC cell lines, we performed a small scale, drug-based screening experiment using YAP cell localization as the read-out. We identified erlotinib as an inducer of YAP cytoplasmic localization, an inhibitor of the TEA luciferase reporter system and the expression of the *bona fide* YAP target gene, Connective Tissue Growth Factor CTGF. On the other hand, BIS I, an inhibitor of PKCδ and GSK3β, caused YAP accumulation into the nucleus. Activation of β-catenin reporter and interfering experiments show that inhibition of the PKCδ/GSK3β pathway triggers YAP nuclear accumulation inducing YAP/TEAD transcriptional response. Inhibition of GSK3β by BIS I reduced the expression levels of SMADs protein and reduced YAP contribution to EMT. Notably, BIS I reduced proliferation, migration and clonogenicity of PDAC cells *in vitro*, phenocopying YAP genetic down-regulation. As shown by chromatin immunoprecipitation experiments and YAP over-expressing rescue experiments, BIS I reverted YAP-dependent EMT program by modulating the expression of the YAP target genes *E-cadherin*, *vimentin*, *CTGF* and of the newly identified target, *CD133*. In conclusion, we identified two different molecules, erlotinib and BIS I, modulating YAP functionality although *via* different mechanisms of action, with the second one specifically inhibiting the YAP-dependent EMT program in PDAC cell lines.

## INTRODUCTION

The Yes-associated protein, YAP, is a transcriptional co-activator containing a proline-rich region responsible for the interaction with SH3 domains of c-Yes and many other proteins [1]. Multiple post-translational modifications (PTMs) regulate the functions of YAP. The Hippo signaling pathway, initially defined as a tissue growth and organ size regulator in *Drosophila*, is a kinase cascade able to negatively regulate YAP localization and activity, by phosphorylating YAP at Serine127. Phosphorylation of YAP by the Hippo pathway leads to its accumulation in the cytoplasm and, by interaction with 14–3–3 proteins, YAP is degraded by a ubiquitination-dependent proteasomal process. Therefore, the Hippo pathway negatively regulates YAP functionality and presence in the nucleus by modulating its cell distribution and its protein expression levels too. Importantly, the Hippo pathway-induced phosphorylation of YAP rules its functionality according to cell density. At low density, YAP is predominantly localized in the nucleus while YAP translocates to the cytoplasm at high cell density [2]. Cytoplasmic YAP has been found associated with numerous protein complexes that mainly mediate its sequestration and consequent functional inactivation. As an example, Angiomotins recruit YAP to Tight Junctions or the actin cytoskeleton, in a Hippo pathway-independent manner, resulting in reduced YAP nuclear localization [3,4]. On the same line, when the WNT pathway is off, the association of YAP with beta-catenin leads to reciprocal inhibition of both proteins [5–7]. GSK3β inhibition by 6-bromoindirubin-30-oxime (BIO) promotes the activation of YAP *via* de-activation of the Hippo pathway [6]. Nuclear localization of YAP protein is associated with its co-transcriptional activity. However, YAP is at the crossroad of many signaling pathways, where it plays a role depending on the upstream stimuli and the binding to its multiple targets. Among the transcription factors bound to YAP, members of the TEAD family were found to be critical partners of YAP in the regulation of gene expression. CTGF has been identified as a direct target gene of YAP-TEAD in mammalian cells, and is crucial in mediating the growth-stimulating and oncogenic function of YAP-TEAD complex [8], but its transcriptional expression depends on the contribution from other YAP interacting transcription factors such as SMADs [9]. Additionally, many other transcription factors have been found associated with YAP such as p73 [10], showing that YAP can mediate oncosuppressive gene expression program according to the cell context. Several pieces of evidence support an important role of YAP in different types of cancer [11,12], pancreatic ductal adenocarcinoma (PDAC) included [13,14]. Indeed, YAP expression, *via* immunohistochemistry studies in pancreatic tumor tissues, was reported as moderate to strong in the nucleus and cytoplasm of the tumor cells compared to adjacent normal tissues. In cell lines, YAP localization was modulated by cell density and its genetic ablation led to a decrease of growth in soft agar of pancreatic cancer cells [12,13]. In PDAC mouse models, YAP has been shown to be an essential promoter of mutant KRAS oncogenic program, specifically inducing the expression of secreted factors as CTGF and CYR61 [15] and associating with FOS to regulate the expression of Epithelial to Mesenchymal Transition genes as *E-cadherin*, *SLUG*, *SNAIL* and *Vimentin* [16]. These pieces of evidence suggest a role of YAP in pancreatic cancer development, potentially playing an important role in the Epithelial to Mesenchymal Transition (EMT) of pancreatic cancer cells. Therefore, the identification of inhibitors of YAP activity could be suitable as a new therapeutic option for PDAC treatment.

However, an intricate network of signaling pathways contributes to EMT in PDAC. TGFβ signaling pathway is frequently genetically altered in PDAC [17], and the “late TGFβ signature” [18] actively promotes late EMT also cooperating with YAP [9] and activating the RAS-ERK pathway promoting the expression of EMT transcription factors such as SNAIL and ZEB1 [19]. CD133 is a well-known cancer stem marker [20] which has been included to the plethora of genes responsible for EMT promotion by activating SRC pathway [21–23].

We performed a small-scale high-content screening for the identification of compounds able to interfere with YAP localization and functionality. This approach allowed us to assign to the widely used Receptor Tyrosine Kinase (RTK) Inhibitor, erlotinib, the ability to sequester YAP into the cytoplasm blocking its co-transcriptional function. Additionally, we found that a small molecule, GF 109203X (BIS I), induces YAP nuclear accumulation and activation, however, modulating its co-transcriptional activity by blocking the YAP-dependent EMT program downregulating SMAD2/3.

## RESULTS

### YAP regulates anchorage-independent growth in PDAC cell lines

**Figure 1:**
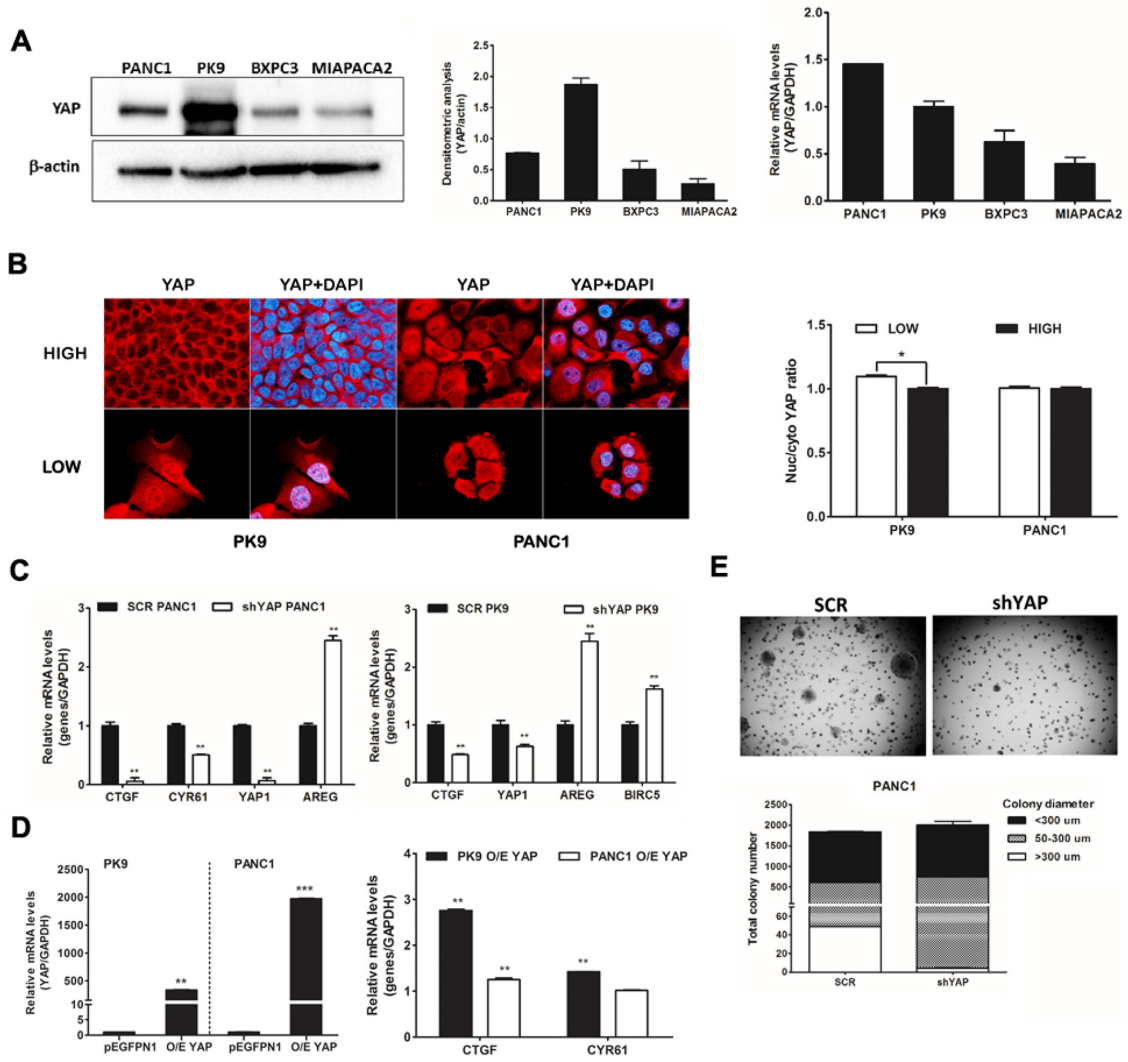
Importance of YAP in PDAC cell lines **A. YAP is expressed in PDAC lines at different levels.** Western blot analysis of endogenous level of YAP in PDAC cell lines and qRT-PCR analysis of YAP mRNA expression. The relative intensity of the bands (left) and YAP mRNA level (right) are shown. **B. Localization of YAP is regulated by cell density in PK9.** PK9 and PANC1 cells were cultured sparsely (LOW) and densely (HIGH) onto glass cover slides (Left panel) and in 96 well-plate (right panel) for 48H. Cells were fixed and nuclei were counterstained with DAPI. The localization of YAP was visualized using a Zeiss Observer Z1 microscope equipped with Apotome module, with a Plan Apochromatic (63X, NA 1.4) objective. Images were acquired using Zen 1.1 (blue edition) imaging software (Zeiss) and assembled with Adobe Photoshop CS3 (Left panel). Quantitative analysis of sub-cellular localization of YAP was quantified using Operetta instrument and Harmony 3.5.2 software. Ratio of YAP Nuc/Cyto is shown. (*p<0.05). **C. YAP functional ablation down-regulates *CTFG* and *CYR61* but not *AREG* and *BIRC5* mRNA levels.** PANC1 (left) and PK9 cells (right) were stably transduced with a lentiviral vector encoding shRNA targeting YAP or a non-targeting control shRNA (SCR). After stable selection with puromycin, the relative levels of endogenous *YAP* and its target genes, *CTGF*, *CYR61*, *AREG* and *BIRC5* mRNA were measured by qRT-PCR (mean±SD). (**P<0.0, **P<0.01 versus SCR). **D. Overexpression of YAP increases *CTGF* and *CYR61* levels in PK9 cells.** PK9 and PANC1 cells were transiently transfected with pEGFP-YAP or empty vector (pEGFPN1) for 24H. Overexpression of YAP was confirmed by qRT-PCR analyses. The expression levels of *CTGF* and *CYR61* were then evaluated. **E. YAP functional ablation attenuates anchorage-independent growth in soft agar.** PANC1 cells were stably transduced with a lentiviral vector encoding shRNA targeting YAP or a non-targeting control shRNA (SCR). These clones (1.5×10^4^ cells) were seeded in 0.35% agar (top agar) medium in 6 well-plates coated with 0.7% agar (based agar) for 2 weeks. Total colony number and colony diameter were measured using Operetta and Harmony 3.5.2 software (below).

We measured the expression level of YAP in a panel of four PDAC cell lines using western blotting and qRT-PCR: PANC1 and PK9 exhibited moderate to high YAP protein levels, respectively, in comparison to BXPC3 and MIAPACA2 cells (Figure 1A). Cell density regulates phosphorylation and localization of YAP *via* the Hippo signaling pathway. High cell density predicts a cytoplasmic YAP localization while YAP appears mainly localized in the nucleus in sparse cell culture of breast cancer cells [24]. We investigated whether cell density regulates YAP localization in pancreatic cancer cells. We assessed the expression level and localization of YAP at different cell densities using immunofluorescence in PK9 and PANC1 cells. Sub-cellular distribution of YAP protein was equivalent in both cases with PANC1 cells, but YAP significantly shuttled from nucleus to the cytoplasm at high cell density in PK9 cells, as determined by high content imaging analysis (Figure 1B). To investigate the functional role of YAP, we interfered YAP expression in PK9 and PANC1 cells using lentiviral transduction of specific shRNA (Supplementary Figure S1A). shYAP-PANC1 and shYAP-PK9 cells showed a decrease of 90% and 40% of YAP mRNA compared to (SCR) control cells, respectively (Figure 1C). *CTGF* and *Cyr61* mRNA expression, *bona fide* YAP targets, were significantly reduced in shYAP-PANC1 and shYAP-PK9 (Figure 1C), whereas other targets like *AREG* and *BIRC5* were distinctly up-regulated in silenced cells, indicating a transcriptional impact due to YAP modulation. On the other hand, *CTGF* expression was found increased in the case of YAP overexpression (O/E) both in PK9 and in PANC1. *CYR61* expression was increased in PK9 O/E YAP. (Figure 1D). Phenotypically, both YAP stable silencing (shYAP) and its transient functional ablation inhibited anchorage-independent growth of PANC1 cells in soft agar (Figure 1E) and slowed their proliferation rate (Supplementary Figure S1B), in good agreement with previous data [14, 25]. Therefore, in PDAC cell lines cultured at high-density, YAP is partially redistributed in the cytoplasm, it has a transcriptional effect controlling the expression of known target genes, it regulates proliferation and the ability of PDAC cells to grow in anchorage-independent conditions.

### Identification of modulators of YAP localization

**Figure 2:**
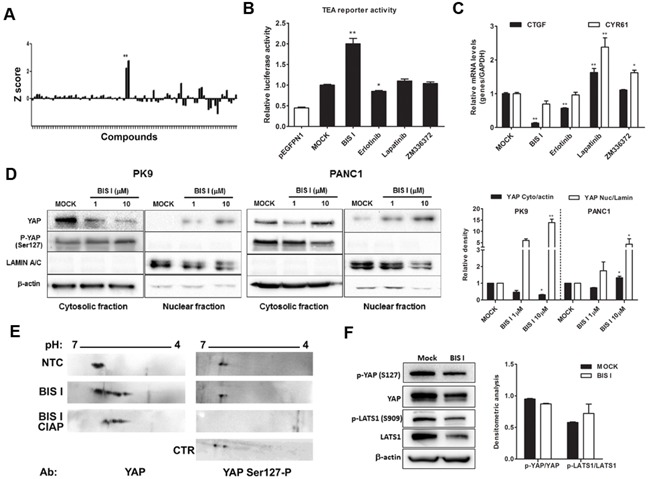
Identification of modulators of YAP localization **A. High-content screening evaluating YAP localization.** A kinase inhibitor's library was administrated to PK9 cells using a high-throughput approach (1μM, 24H). The ratio between nuclear and cytoplasmic regions was calculated and normalized to untreated controls. The Z-score was reported in a graph, positive and negative values indicate nuclear accumulation and cytoplasmic localization, respectively. Fixed cells were incubated with antibody against YAP and DAPI staining. Sub-cellular localization of endogenous YAP protein was detected by Operetta and analyzed with Harmony 3.5.2 software. **B. Modulation of TEA reporter by hit compounds.** PK9 cells were transiently co-transfected with pEGFPN1 or YAP, TEA reporter (8xGTIIC-Luc reporter), and *Renilla* luciferase to record YAP/TAZ-dependent transcriptional activity. Cells were then treated with different compounds for 24 H and the firefly luciferase signals were normalized to the ones of *Renilla* luciferase. Data are globally normalized to MOCK and are presented as mean±SD. **C. Modulation of *CTGF* and *CYR61* by hit compounds.** The panel represent qRT-PCR for YAP/TAZ target genes *CTGF* and *Cyr61*, relative to *GAPDH* expression. PK9 cells were treated with 5μM of different compounds and data, normalized to MOCK, are presented as mean±SD. (*p<0.05 and **p<0.01). **D. BIS I induces YAP nuclear accumulation.** Western blot analysis of nuclear fraction and cytosolic fraction of YAP in PK9 and PANC1 cell lines after treatment with 1μM and 10μM of BIS I for 24H. The relative intensities of the bands are also shown (right). Data are normalized to MOCK and presented as mean±SD. (*p<0.05 and **p<0.01). **E. BIS I induces YAP post-translational modifications.** Filters were blotted with antibodies against YAP and YAP Ser127-P. As negative control for phosphorylation the treated sample was incubated with calf intestinal alkaline phosphatase (CIAP). As positive control for phosphorylation at Ser127, cell lysates from high density culture was used. **F. BIS I does not affect the Hippo pathway.** Western blot analysis of an upstream regulator of YAP, LATS1 and its phosphorylated form (Ser909). PK9 cells were treated with BIS I 10μM for 24 H. Phosphorylation of YAP and LATS1 was measured by western blot. The relative intensities of the bands were normalized to β-actin levels (right).

To gain further insight into the molecular players regulating YAP localization, we performed a small-scale nucleo-cytoplasmic high content assay to quantify YAP protein subcellular localization in PDAC cells. As a cell model, we used PK9 cells as YAP was re-localizing into the cytoplasm at high cell density (Figure 1B). We used a library of 80 characterized kinase inhibitors (see methods) with the aim to find molecules that could modulate accumulation of YAP in the cytoplasm or the nucleus, to identify the signaling cascade responsible for these subcellular re-localizations and the biological effects caused by its sub-cellular re-distribution. Most of the molecules did not affect YAP localization being the Z-score values of the nuclear/cytoplasmic intensity close to controls (Figure 2A, Table 1, Supplementary Table S1). Few compounds were further increasing cytoplasmic YAP compared to control, and only two compounds led to significant YAP accumulation into the nucleus. Interestingly, first hits among YAP cytoplasmic accumulators were inhibitors of tyrosine kinase receptors (RTKIs) such as Genistein and Tyrphostins and one inhibitor of the RAS pathway as ZM336372. On the other hand, inducers of YAP nuclear shuttling were BIS I and Ro 31-8220, two representatives of the bisindolylmaleimide family of Ser/Thr kinase inhibitors, as PKCs (Table 1).

**Table 1:** Hits from High-Content Screening

Z-score	Molecule	Molecular Target
	YAP Nuclear Accumulators	
0.915	GF 109203X	PKC
0.971	Ro 31-8220	PKC
	**YAP Cytoplasmic Accumulators**	
1.382	Tyrphostin AG 1295	Tyrosine kinases
1.355	ZM 336372	cRAF
1.238	Genistein	Tyrosine Kinases
1.182	N9-Isopropyl-olomoucine	CDK
1.18	SP 600125	JNK
1.171	AG-1296	PDGFRK
1.169	Kenpaullone	GSK3β
1.132	AG-494	EGFRK, PDGFRK

Lower values indicate higher nuclear accumulation, higher values indicate higher cytoplasmic accumulation

### Cytoplasmic inducers marginally modulate YAP co-transcriptional activity

As the receptor tyrosine kinase inhibitors identified belong to the family of tyrphostin [26], precursors of the more potent and clinically used inhibitors of RTKIs, such as erlotinib and lapatinib, we decided to use these two drugs for further investigation. Moreover, given the importance of the constitutive activation of the RAS pathway in PDAC [27], we also evaluated the efficacy of ZM336372 in inhibiting YAP-dependent transcriptional effects by using TEA luciferase reporter system as a readout. Only erlotinib inhibited modestly, but significantly, TEA reporter and reduced the expression level of *CTGF* but with no efficacy on *Cyr61* (Figure 2B, 2C). On the contrary, lapatinib and ZM336372 increased *CTGF* and *Cyr61* expression levels (Figure 2C). Summing up, only erlotinib showed a minimal, although significant, negative modulation of YAP co-transcriptional activity.

### BIS I changes YAP co-transcriptional activity and inhibits anchorage independent growth

**Figure 3:**
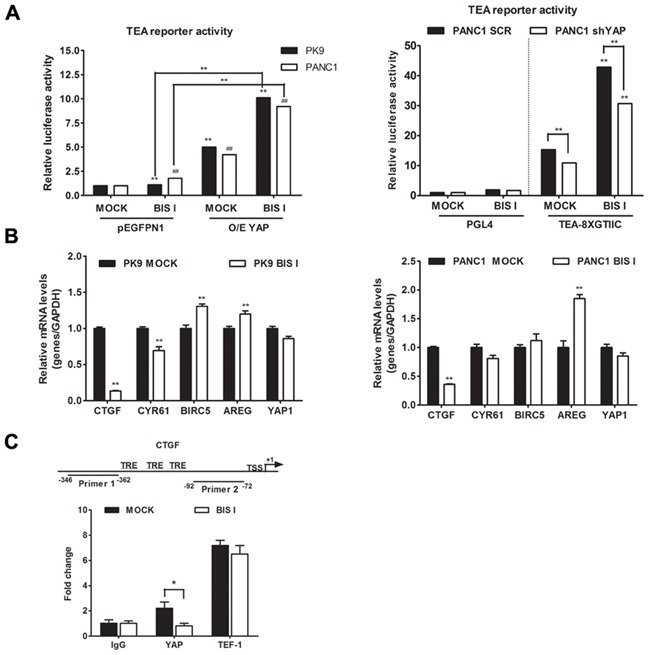
BIS I treatment phenocopies YAP functional ablation **A. BIS I modulates TEA reporter in YAP-dependent manner.** Left panel: BIS I activates TEA reporter activity. PK9 and PANC1 cells were transiently co-transfected with YAP (O/E YAP) or without YAP (pEGFPN1) and TEA reporter (8xGTIIC-Luc reporter), then treated with BIS I 5μM for 24H. Right panel: YAP is required for TEA reporter activation. Stably YAP silenced PANC1 cells were co-transfected with TEA reporter (8xGTIIC-Luc reporter) or its empty vector (pGL4), and *Renilla*. The firefly luciferase signals were normalized to the ones of *Renilla.* (mean±SD from biological triplicates) (*p<0.05 and **p<0.01 versus MOCK of PK9 and #p<0.05 and ##p<0.01 versus MOCK of PANC1). **B. BIS I modulates YAP target genes.** PK9 (left panel) and PANC1 (right panel) cells were treated with 5μM BIS I for 24H. Quantitative RT-PCRs of *CTGF*, *Cyr61*, *BIRC5*, *AREG*, YAP/TAZ target genes and *YAP* relative to *GAPDH* expression with respect to MOCK are presented as mean±SD. (*p<0.05 and **p<0.01 versus MOCK). **C. BIS I displaces YAP from CTGF promoter.** Map of CTGF promoter region with positions of the two primers used for ChIP analysis. TSS indicates the transcription start site, while TRE indicate the previously identified TEAD responsive elements. Chromatin immunoprecipitation (ChIP) at CTGF promoter was performed using antibodies against YAP, TEF-1 and IgG as negative control. After DNA extraction and qRT-PCR, results were normalized to non immunoprecipitated sample (INPUT) and compared to IgG for statistical significance. BIS I was able to reduce the DNA enrichment observed for YAP, whereas it was ineffective against TEF-1 DNA-binding protein.

**Figure 4:**
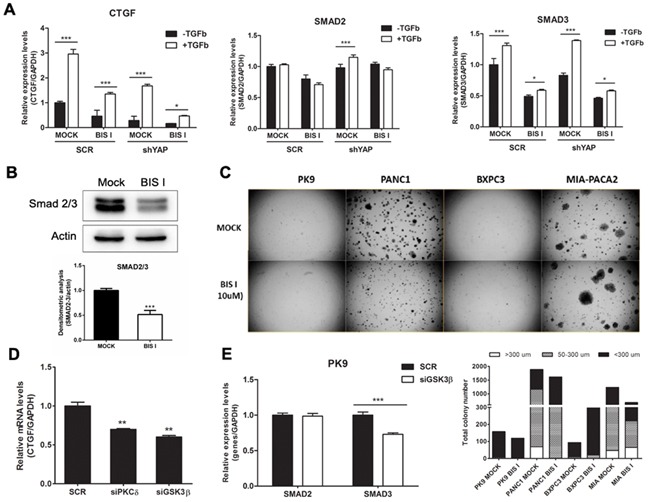
The CTGF expression level was modulated by the TGF-β and Hippo pathways in PDAC **A. BIS I inhibits TGF-β induced *CTGF* expression and reduces *SMAD2/3* gene expression levels.** PK9 cells were stably transduced with a lentiviral vector encoding shRNA targeting YAP (shYAP) or a non-targeting control shRNA (SCR). They were then treated with BIS I 5μM in the presence and absence of TGF-β 50ng/ml for 24H. The expression levels of *CTGF, SMAD2* and *SMAD3* were then evaluated. (*p<0.05 and ***p<0.001 versus MOCK). **B. BIS I down-regulated Smad2/3 protein levels.** PK9 cells were seeded and treated with BIS I 10μM for 24H. The endogenous protein level of Smad2/3 was evaluated by western blotting against Smad2/3 antibody. The relative intensities of the bands normalized by β-actin are shown below. (***p<0.001 versus MOCK). **C. BIS I inhibits anchorage-independent growth of PDAC.** PDAC (1.5×10^4^ cells) were seeded on 0.35% agar (top agar) culture medium in 6 well-plated coated with 0.7% agar (based agar). Cells were treated with BIS I 10μM for 2 weeks. Total colony number and colony diameter were measured using Operetta instrument. **D. The CTGF expression level was modulated by PKCδ and GSK3β in PK9 cells.** PK9 cells were incubated with siRNA targeting PKCδ, GSK3β, and non-targeting control (SCR) for 72H. Quantitative RT-PCRs of *CTGF* relative to *GAPDH* expression with respect to SCR are presented as mean+SD. (*p<0.05 and **p<0.01 versus SCR). **E. Genetic ablation of GSK3β suppresses SMAD expression levels in PK9.** PK9 cells were incubated with siRNA targeting GSK3β, and non-targeting control (SCR) for 72H. Quantitative RT-PCRs of *CTGF* relative to *GAPDH* expression with respect to SCR are presented as mean+SD. (***p<0.01 versus SCR).

Bisindolylmaleimide chemical family of compounds are strong inhibitors of several kinases in the nanomolar range and, therefore, it is difficult to associate a molecular target directly to its efficacy. BIS I is a cell-permeable and reversible inhibitor of protein kinases C (PKCs) both conventional and atypical, but also of GSK3β [28]. Moreover, compounds of the same class show differential selectivity towards the same serine/threonine kinases. For example, Go9676 was reported to be more specific for PKCα than for PKCδ [29], while BIS I behaves oppositely [30]. Since bisindolylmaleimides are fluorescent compounds and could have interfered with the immunofluorescence-based screening, we performed nuclear/cytoplasmic separation and Western blots analyzes of YAP levels upon drug treatments. We observed that BIS I induced YAP nuclear localization in PDAC cell lines, confirming the indication coming from the HCS experiment (Figure 2D). Then we evaluated the stability of YAP protein during BIS I treatment, and, in the time frame of 48 hours, YAP protein was stable, suggesting that we observed a pure subcellular re-localization not affected by changes in protein expression level (Supplementary Figure S1C). During re-localization, the YAP post-translation status was deeply changed, as many phosphorylation spots were present in the two-dimensional western blots (Figure 2E). Hippo signaling pathway was not directly involved in the observed PTMs because the phosphorylation state of YAP-S127 and LATS-S909 did not change (Figure 2F). Additionally, we observed a decrease in the expression level of LATS protein, an effect that is likely independent of the activation of the Hippo signaling but might contribute to the translocation of YAP into the nucleus. BIS I amplified TEA reporter signal in the basal conditions in PANC1 cells and during YAP overexpression both in PK9 and PANC1 cells (Figure 3A) and induced TEA reporter signal reduction during functional ablation of YAP. Therefore, the effect of BIS I on TEA reporter depends on the presence of YAP (Figure 2B, 3A). Unexpectedly, BIS I significantly suppressed *CTGF* and *Cyr61* mRNA expression and slightly increased *AREG* and *BIRC5* mRNA expression in PK9 and PANC1 cells at 24 hours of treatment, mimicking YAP ablation (Figure 3B). To evaluate if BIS I displaces YAP from *CTGF* promoter, despite YAP presence in the nucleus, we performed chromatin immunoprecipitation of YAP and evaluated the amplification of the *CTGF* promoter [31]. Indeed, YAP was no more associated with the *CTGF* promoter during BIS I treatment, but TEAD1 was still present (Figure 3C). Additionally, we observed that BIS I inhibited TGF-β induced *CTGF* expression in a YAP independent manner but activated TEA reporter in a TGF-β independent manner (Figure 4A and Supplementary Figure S1D). BIS I treatment reduced the expression level of *SMAD2/3* mRNAs and proteins in a YAP independent manner (Figure 4A, Figure 4B, Supplementary Figure S1E). BIS I phenocopied the effects of YAP functional ablation: it slowed down cell proliferation and induced an accumulation in the S-phase (Supplementary Figure S1B, S1F). Most importantly, BIS I was readily effective in reducing the anchorage-independent growth of PDAC cell lines. Among the four cell lines tested, PANC1 and MIAPACA2 formed colonies within two weeks, while PK9 took longer and we could not detect BXPC3 colony formation. BIS I reduced the total number of colonies of PANC1, MIAPACA2, and PK9 and reduced the colony dimensions of PANC1 and PK9 but increasing MIAPACA2 ones (Figure 4C). Summing up, BIS I induced YAP into the nucleus and triggered TEAD response; however, it inhibited anchorage-independent cancer cell growth and proliferation, phenocopying YAP ablation and inhibiting TGFβ-dependent cell response by decreasing the expression of SMADs and SMAD/YAP co-regulated genes.

### CD133 gene expression is regulated by YAP and inhibited by BIS I

**Figure 5:**
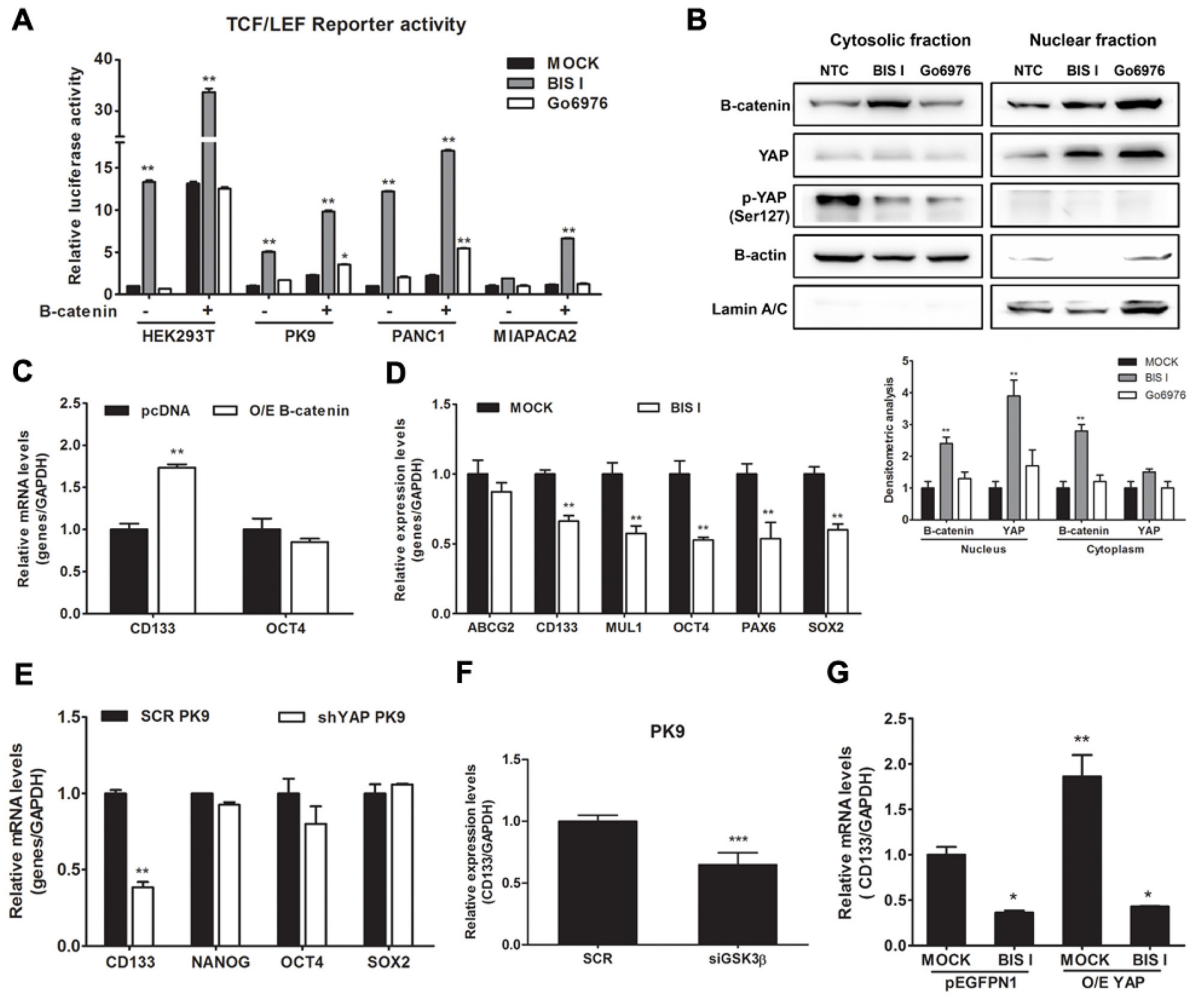
BIS I activates β-catenin and downregulates the expression level of cancer staminality genes **A. BIS I activates β-catenin signaling pathway.** β-catenin induced activation of TOP-flash (TCF/LEF) luciferase reporter was performed in HEK293T and PDAC cells lines. Cells were co-transfected with TOP-flash luciferase reporter and in the presence and absence of β-catenin. BIS I and Go6976 5μM were used for 24H treatment after transfection. Data are presented as average fold induction relative to MOCK. (*p<0.05 and **p<0.01). **B. BIS I modulates β-catenin nuclear localization.** Western blot analysis of nuclear fraction and cytosolic fraction of β-catenin in PK9 cells after treatment with BIS-I and Go6976 10μM for 24H. The relative intensities of the bands are shown below. The relative intensities of the bands was normalized by β-actin and lamin A/C for cytosolic and nuclear protein levels, respectively. **C. β-catenin regulates CD133 expression.** PK9 cells were transiently transfected with indicated β-catenin plasmid for 24H. Quantitative RT-PCRs of *CD133* and *OCT4* relative to *GAPDH* expression with respect to MOCK are presented as mean±SD. (*p<0.05 and **p<0.01). **D. BIS I inhibits expression of staminality markers in PK9.** PK9 cells were treated with BIS I 5μM for 24H and expression levels of *ABCG2*, *CD133*, *MUL1*, *OCT4*, *PAX6*, and *SOX2* were analyzed by qRT-PCR relative to *GAPDH* expression. Data are presented as mean±SD. (**p<0.01 versus MOCK). **E. Genetic ablation of YAP down-regulates CD133 expression.** Stemness markers were measured by qRT-PCR in stably YAP silenced PK9 cells. Data are presented as mean±SD. (**p<0.01 versus SCR). **F. Genetic ablation of GSK3β down-regulates CD133 expression.** PK9 cells were incubated with siRNA targeting GSK3β, and non-targeting control (SCR) for 72H. Quantitative RT-PCRs of *CD133* relative to *GAPDH* expression with respect to SCR are presented as mean±SD. (***p<0.01 versus SCR). **G. BIS-I treatment reverts the CD133 up-regulation induced by YAP overexpression.** PK9 cells were transiently co-transfected with indicated plasmids (YAP and pEGFPN1) and in the presence and absence of BIS I treatment for 24H. *CD133* expression was measured by qRT-PCR. Data are presented as mean±SD. (*p<0.05 and **p<0.01 versus MOCK).

To further investigate the molecular mechanism of bisindolylmaleimides leading to YAP nuclear accumulation, we evaluated four of them for their ability to modulate *CTGF* expression levels. BIS I and BIS II reduced *CTGF* expression in PK9 and PANC1 cells, although to a different extent, while Go9676 increased *CTGF* expression. BIS IV showed an increase of *CTGF* expression only in PK9 cells with no activity in the other cell lines (Supplementary Figure S2A). None of the bisindolylmaleimides tested showed toxic effects (Supplementary Figure S2B). Given the relative specificity of BIS I for PKCδ/GSK3β, while Go9676 is more specific for PKCα, we hypothesized that PKCδ/GSK3β ablation could phenocopy BIS I treatment. Transient silencing of PKCδ and GSK3β showed that *CTGF* decreased in both cases linking the activity of these two kinases in *CTGF* expression regulation (Figure 4D, Supplementary Figure S2C). Short-term treatment with the GSK3β inhibitor LiCl induced the expression of *CTGF* mRNA, but long-term treatment decreased *CTGF* expression [32]. Interestingly, the downregulation of *SMAD2* mRNA occurred only at a late time point whereas *SMAD3* was already down-regulated at 6 hours (Supplementary Figure S2D). BIS I induced the downregulation of both *SMADs* already at 6 hours. Silencing of GSK3β activated TEA reporter only in the presence of YAP overexpression, similarly to BIS I treatment (Supplementary Figure S2E) and reduced the expression of *SMAD3* mRNA (Figure 4E) as BIS I treatment. Since the association of YAP to the destruction complex has been reported [5], we evaluated the activation of the WNT/β-catenin signaling in PDAC cell lines during BIS I treatment. We performed a reporter assay with a construct expressing luciferase under the control of tandem repeats of TCF binding site (TOPFlash) or a mutated one (FOPFlash). BIS I strongly activated WNT/β-catenin reporter activity in all of the PDAC cell lines used and in HEK293T cells (Figure 5A, Supplementary Figure S2F) as well as induced the accumulation of β-catenin in the nucleus (Figure 5B). Coherently, Go9676 did not activate TCF/LCF reporter nor induced β-Catenin into the nucleus (Figure 5A, 5B).

Given the importance of YAP and β-Catenin in regulating differentiation, we investigated if BIS I could affect the expression levels of stemness markers of PDAC. In our case, pancreatic stemness gene *CD133* was up-regulated upon β-Catenin over-expression (Figure 5C). These stemness genes were down-regulated during BIS I treatment (Figure 5D), but, interestingly, only *CD133* was affected by YAP and GSK3β ablation (Figure 5E and 5F). Moreover, YAP overexpression induced the up-regulation of *CD133* that was blocked by BIS I (Figure 5G), therefore identifying *CD133* as a new gene regulated by YAP and GSK3β. To sum up, BIS I induced YAP and β-Catenin nuclear accumulation by inhibiting the PKCδ/GSK3β pathway. Moreover, BIS I treatment decreased the level of cancer stem cell markers, but only the effect on *CD133* could be ascribed to a loss of YAP functionality. Notably, this gene is a new critical regulator of EMT in PDAC [21,23].

### YAP-dependent EMT transcriptional program is inhibited by BIS I

**Figure 6:**
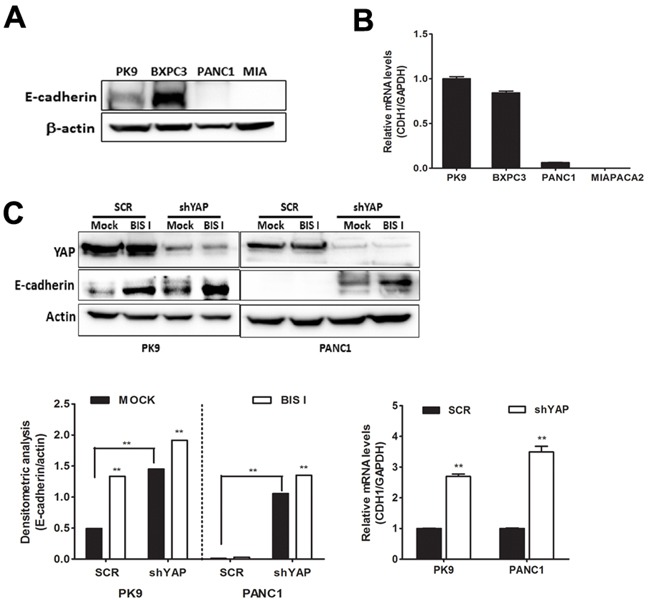
BIS I reverts YAP-induced EMT in PDAC cell lines **A. Endogenous protein level of E-cadherin in PDAC cell lines.** Western blot analysis of endogenous level of E-cadherin in PDAC cell lines. **B. Expression level of E-cadherin mRNA in PDAC cell lines.** qRT-PCR analysis of *CDH1* mRNA expression was performed in PDAC cell lines. **C. Both genetic ablation of YAP and BIS I treatment induce E-cadherin expression levels.** SCR or stably YAP-silenced PK9 and PANC1 cells were treated with BIS I 5μM for 24H. Western blot analysis of endogenous level of YAP and E-cadherin was performed. The relative levels of endogenous E-cadherin protein (left) and mRNA levels from these lysates (right) were evaluated by immunoblot and qRT-PCR, as shown below.

**Figure 7:**
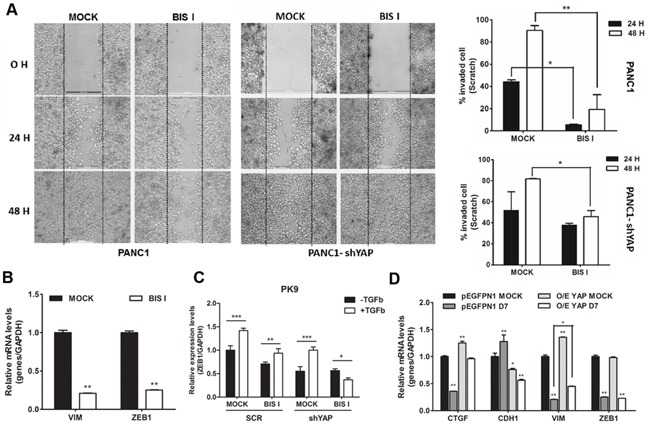
Genetic ablation of YAP and BIS I treatment regulate cell migration **A. BIS I reduces cell migration synergizing with YAP silencing.** Scratch assay was performed in SCR or stably YAP-silenced PANC1 cells. Images of invaded cells at 0, 24, and 48 H after scratching and treatment with BIS I were taken from a time-lapse sequence of PANC1 cell migration; wounds with consistent shape within each well were generated using 200 μl tip. Percentage of invaded cells at different time point is indicated (right panels) as calculated by ImageJ softwre. (*p<0.05 and **p<0.01). **B. BIS I down-regulates *VIM* and *ZEB1* mRNAs.** PK9 cells were treated with BIS I for 24H. Expression level of *VIM* and *ZEB1* were measured by qRT-PCR. Data are presented as mean±SD. (**p<0.01 versus MOCK). **C. BIS I inhibits TFG-β induced ZEB1 expression.** PK9 cells were stably transduced with a lentiviral vector encoding shRNA targeting YAP (shYAP) or a non-targeting control shRNA (SCR). They were then treated with BIS I 5μM in the presence and absence of TGF-β 50ng/ml for 24H. The expression levels of *ZEB1* was evaluated. (*p<0.05, **p<0.01 and ***p<0.001). **D. Overexpression of YAP reverts the effect of BIS-I on the expression of *CTGF*, *CDH1* and *VIM*.** PK9 cells were transiently co-transfected with YAP and pEGFPN1 plasmids and treated with BIS I for 24H. *CTGF*, *CDH1*, *VIM*, and *ZEB1* expression levels were measured by qRT-PCR. Data are presented as mean±SD. (*p<0.05 and **p<0.01).

EMT signature, in PDAC, in mainly driven by the activation of the RAS pathway in association with the transcriptional program induced by FOS, SMADs, and YAP [16] and the loss of E-cadherin is a major event during EMT [22]. Our cell lines exhibit different expression levels of E-cadherin (Figure 6A, 6B). PK9 and BxPC3 cell lines showed high protein level of E-cadherin whereas PANC1 and MIAPACA2 had low to undetectable E-cadherin protein levels (Figure 6A). This expression profile of E-cadherin in PDAC is consistent with its mRNA levels (Figure 6B). Our results show that endogenous levels of E-cadherin are inversely correlated with the anchorage-independent growth ability of these PDAC cell lines (Figure 4C). Importantly, EMT signature was dependent on YAP as, in shYAP-PK9 and shYAP-PANC1, E-cadherin and CD133 expression was reverted (Figure 6C, Figure 5E, 5G). During BIS I treatment, the clonogenic and migration abilities of PDAC, markers of EMT, were completely inhibited (Figure 4C, 7A). BIS I was able to revert the EMT signature by restoring E-Cadherin expression, as well as by regulating other EMT markers as *vimentin*, *ZEB1* and *CD133* also in the presence of TGFβ (Figure 5G, 7B, 7C, 7D). Migration of PANC1 cells decreased by administration of BIS I in sh-YAP PANC1 cells at 48 hours (Figure 7A). More notably, the effect of BIS I on the modulation of *CTGF, E-cadherin, vimentin,* and *CD133* was rescued by YAP overexpression showing that BIS I mechanism of action relies on the inhibition of the EMT-related, YAP-dependent transcriptional program (Figure 5G, 7D). In sum, we show that BIS I inhibits EMT in PDAC cell lines triggering the expression of epithelium markers by down-regulating SMAD2/3 and blunting YAP co-transcriptional activity.

## DISCUSSION

In this study, we show that BIS I, an inhibitor of the PKCδ/GSK3β pathway, reverts the EMT transcriptional program in PDAC cell lines inhibiting the TGFβ pathway and de-potentiating YAP contribution to EMT *via* down-regulation of SMAD2/3.

In agreement with previous reports [14,33,34], we observed that, in PDAC cell lines, YAP mainly regulates anchorage-independent growth, migration, and proliferation. However, YAP governs the expression of only some of the *bona fide* YAP target genes in these cell lines as only *CTGF* and *CYR61*, but not *AREG* or *BIRC5*, decreased during YAP functional ablation. Indeed, in a KRAS mutant context, YAP, being post-translationally modulated by KRAS/MAPK signaling cascade, promotes the expression of pro-proliferative secretory factors as *CTGF* and *CYR61*, preferentially [15]. From our small-scale screening of kinase inhibitors, using as reporter system the localization of the endogenous YAP protein, we found out that general inhibitors of RTKs and one RAS inhibitor induced YAP accumulation into the cytoplasm. This finding suggests a functional link between the EGFR and RAS pathway and YAP activity as observed in *Drosophila* [35], liver carcinoma [36], NSCLC cells [37] and pancreas itself [16]. Unfortunately, none of these inhibitors was potent enough to inhibit TEA reporter system and decrease *CTGF* expression, suggesting that the residual amount of nuclear YAP was still active. Only erlotinib showed a small but significant trend towards inhibition of YAP co-transcriptional activity. Indeed, erlotinib arrests NRG1-ERBB4-YAP signaling in breast cancer cell lines [38], and suggests a further rationale for the utilization of erlotinib in PDAC [39]. Additionally, we found of interest the behavior of YAP nuclear accumulators, i.e. bisindolylmaleimides. The mechanistic explanation can be linked to the pleiotropic effect of BIS I. One possibility is that BIS I induced arrest of the proteasome leading to accumulation of β-catenin [40, 41] and YAP, being both proteins targeted for degradation. Alternatively, the BIS I induced inhibition of PKCδ/GSK3β activity can lead to the degradation of the destruction complex as proved by β-catenin and YAP nuclear accumulation. Inhibition of the GSK3β by BIS I led to osteogenic differentiation and suppression of adipocyte differentiation by β-catenin stabilization [42, 43]. More importantly, these data are in agreement with the inhibition of the GSK3β by BIO that led to the activation of YAP/TEAD response [6]. We did not observe a clear inactivation of the Hippo pathway but a down-regulation of LATS that explains the nuclear translocation of YAP, also in an independent manner from the association to the destruction complex [6]. Indeed, we observed the activation of the TEA reporter system during BIS I treatment, but, at the same time, a clear, YAP-dependent, decrease of *CTGF* and *CYR61* mRNAs. *CTGF* promoter region is highly regulated, and several transcription factors contribute to the activation of this locus. In mesangial and gingival cells, BIS I blockage of basal and TGFβ-induced regulation of *CTGF* [44,45] was explained by inhibition of GSK3β. Moreover, in hepatocarcinoma cells, PKCδ-mediated TGFβ signaling led to *CTGF* expression by inhibiting the phosphatase PPM1A, which is responsible for the SMAD2/3 inactivation [46]. Downregulation of *CTGF* upon functional ablation of PKCδ and GSK3β highlights that the similar mechanisms occur in PDAC cell lines (Figure 4D) and can be explained by *SMAD3* mRNA downregulation (Figure 4E). GSK3β inhibition by LiCl induces a biphasic *CTGF* expression regulation: an initial burst of expression, coherent with YAP nuclear accumulation, is followed by a long-term downregulation of the gene. This secondary response is likely due to the parallel downregulation of *SMAD2/3* (Supplementary Figure S2D, S2E). BIS I down-regulated *CTGF* and *SMAD2/3* already at 6 hours, therefore showing a strong potency in blocking TGFβ in a *SMAD4* deficient context, a frequent lesion in PDAC [47] and our cell lines. The same mechanism applies to YAP/SMADs co-regulated genes and, given the importance of this association in promoting EMT [48], it explains the specific reversion of EMT markers. Therefore, the down-regulation of SMADs dampens the YAP co-transcriptional activity of EMT genes. Indeed, YAP overexpression rescued phenotypically the ability to migrate and grow in anchorage-independent condition, and, molecularly, it inhibited the re-expression of E-cadherin and blunted the downregulation of *CTGF*, *CD133,* and *Vimentin*. Additionally, we showed that BIS I induced displacement of YAP from *CTGF* promoter, showing a loss of function of nuclear YAP on specific genes, likely driven by the loss of SMADs partner. Finally, we observed that BIS I induced the downregulation of many cancer stem genes, CD133 included, and we found that this new marker of EMT is highly regulated as YAP, GSK3β, and β-catenin modulated CD133 expression, addressing this gene as a co-regulated gene by these three factors. Further experiments are necessary to clarify this point. However, it suggests that inhibition of the SRC pathway *via* dasatinib [23,49] may influence EMT in PDAC by modulating YAP activity.

## MATERIALS AND METHODS

### Antibodies, plasmids and reagents

The following antibodies were used: Anti-YAP (sc-101199), anti TEF-1 (sc-376113), Anti-GSK3β (sc-9166), Anti phospho GSK3β Ser9 (sc-11757), Anti β-catenin (sc-7199) (Santa Cruz Biotechnology). Anti-Lats1 (3477), Anti-phospho Lats1 Ser909 (9157), Anti-Smad2/3 (8685) and Anti-phospho-YAP (S127) (4911), Anti β-actin (3700) (Cell Signaling). Alexa Fluor 488-conjugated secondary antibodies (Invitrogen). The chemicals were used in this study: GF 109203X (B6292), Go6976 (G1171), phorbol 12-myristate 13-acetate (PMA) (Sigma, P1585) were purchased from sigma. Lapatinib (S1028), Erlotinib (S1023) were purchased from Selleckchem. Recombinant human transforming growth factor 1 (TGF-β) (PHG9204) was purchased from Life technologies. The plasmids were used: 8xGTIIC-luciferase (#34615), YAP-GFP (12), pEGFP-N1 (Clontech), pGL4 (Promega), human B-catenin pcDNA3 (#16828), c-Flag pcDNA3 (#20011), TOP flash and FOP flash were gifted from Dr. Arthit [50].

### Cell culture and transfections

Human pancreatic carcinoma cell lines (PK9, MIAPACA2, PANC1, BxPC3) were kindly provided by G. Feldmann [12] and HEK293T were cultured at 37°C in high glucose Dulbecco's modified Eagle's medium (DMEM) supplemented with 10% Fetal bovine serum (FBS) (Invitrogen), 1X vitamin solution (Sigma), 1X non-essential amino-acid solution (NEAA, Biosource), 1X Sodiumpyruvate (Gibco) and 1X Penicillin-Streptomycin (LONZA) in a humidified incubator with 5% CO2. Transfections were carried out in 6 or 24 well-plates using Lipofectamine 3000 kit (Life Techonologies) as described by the manufacturer. siRNAs for knockdown of PKCδ (sc-36253) and GSK3β (sc-35527) and control siRNA (sc-37007) were transfected using INTERFERIN siRNA transfection reagent (Polyplus transfection) according to manufacturer's protocol. After 72 h of incubation, proteins were extracted for analysis by western blot analysis as describe below.

### Lentriviral particles production and stable clones selection

To generate shYAP expressing stable PK9 and PANC1 cells, The pLKO.1-based lentiviral plasmid containing YAP shRNA (NM006106) expression cassettes were purchased from Sigma-Aldrich. Scramble shRNA (Addgene plasmid 1864) was used as a control. Vectors were produced in HEK293T cells by co-transfection of the different transfer vectors with the packaging plasmid pCMV-deltaR8.91 and the VSV envelope-coding plasmid pMD2.G. After transfection (48h), lentiviral supernatant was filtered through a 0.45 μm syringe filter and used to infect YAP into PK9 and PANC1 cells by spinning them down with vector containing supernatants for 90 min at 1500xg at room temperature and leaving them incubate overnight at 37°C, then the fresh medium were replaced the transduction supernatant. Cells were then further incubated for 72H before collection for WB. Stable silent cells were selected using 3 μg/ml puromycin (Sigma) in the culture medium.

### Immunofluorescence staining and drug screening

PK9 and PANC1 cells were cultured sparsely (LOW) and densely (HIGH) onto glass cover slides and in 96 well-plate for 48 H. Cells were fixed and localization of YAP was visualized and nuclei were counterstained with DAPI. Immunofluorescence analysis was performed using a Zeiss Observer Z1 microscope equipped with Apotome module, with a Plan Apochromatic (63X, NA 1.4) objective. Images were acquired using Zen 1.1 (blue edition) imaging software (Zeiss) and assembled with Adobe Photoshop CS3. Drug screening, PK9 cells were seeded on OptiPlate-96 Black, Black Opaque 96-well Microplate (PerkinElmer) at a density of 5000 cells/well, to obtain a 70% confluence at the end of the assay. The library of known kinase inhibitors was re-suspended in DMSO and diluted in PBS 1X. The compounds were administrated at the final concentration of 1μM for 24H. Cells were fixed as previously described [52], primary antibody against YAP (1:500) and secondary fluorophore conjugated (Alexa 488) antibody (1:10000) were diluted in PBS + BSA 0.2%. DAPI (1.5 μg/ml) in PBS + BSA 0.2% was used to detect nuclei. PerkinElmer image plate reader Operetta was used for imaging and evaluation. The ratio between nuclear and cytoplasmic signal represents the mean of single cells for every well and it were normalized to untreated control. The Z score was calculated to evaluate the significance of results from the screening. Z score was calculated as follow: Z = (X–mean)/standard deviation. X = normalized sample ratio [51].

### Soft-agar and cell migration assay

Each 6-well plate was coated with 1 ml of bottom agar (DMEM containing 10% FBS with and 0.7% agar). PDAC cell lines, shYAP expressing stable PANC-1 (1.5×104 cells) were resuspended in 1ml of top agar (DMEM containing 10% FBS with and 0.35% agar) into each well. Cells were treated with 1ml of BIS I and incubated for 2-3 weeks. They were replaced with fresh medium with/without treatment every 3 days. PerkinElmer image plate reader Operetta was used for imaging and colony evaluation.

Cell migration assay. Confluent cells were seeded in 6-well plate and wounded by a 200μl pipette tip. Media were replaced with BIS I treatment. Images of the same field were acquired immediately (0h), after 24 and 48 hours using a Leica DM IL Led microscope (5X magnification). Wounded-open areas were photographed and measured at the time of scratch and 2 days. Relative invaded area was measured using Image-J software.

### Western blot and sub-cellular fractionation

Cells were treated for 24H then they were lysed in cold modified cytosolic lysis buffer (10mM HEPES pH 7.0, 10mM KCl, 1mM EDTA, 0.5% NP-40, 1mM DTT, and Protease inhibitor cocktail). The supernatant containing cytosolic fraction was collected by centrifugation at 3000xg for 5 minutes at 4°C. Nuclear pellets were then re-suspended in cold modified nuclear lysis buffer (10mM HEPES pH 7.0, 400mM NaCl, 1mM EDTA, 25% Glycerol, 1mM DTT, and Protease inhibitor cocktail). The nuclear extract was harvested by centrifugation at 12000xg for 15 minutes at 4°C. Equal quantities of proteins were separated by electrophoresis on a 12% SDS-page gels. The blots were incubated with YAP1, Phospho-YAP-1 (Ser127), β-catenin antibodies overnight at 4°C. β-actin and Lamin A/C served as the loading controls for the cytosolic and nuclear fraction, respectively.

Luciferase reporter assay. HEK293T, PK9 and PANC1 were seeded in 24 well-plates then indicated plasmids were co-transfected using Lipofectamine 3000 (Invitrogen) or TransIT-LT1 Transfection Reagent (Mirus). After transfection (24H), cells were treated with BIS family compounds including GF 109203X or BIS I (5μM), Go6976 (5μM), Erlotinib (5μM), Lapatinib (5 μM), and PMA (1 μM) for 24H. Cells were lysed and luciferase activity was assayed using the enhanced luciferase assay kit (Promega) following the manufacturer's instructions. The firefly luciferase activity levels were measure and normalized to Renilla luciferase activity.

### RNA isolation, real-time PCR and ChIP assay

Total RNA was extracted using RNA isolation Mini Kit (Agilent Technologies and successively treated with RNA-free DNase. RNA was subjected for reverse transcription (RT) with iScript reverse transcriptase (Bio-rad). cDNA was then diluted and 50ng total of cDNA was used for qRT-PCR with gene-specific primers using KAPA SYBR FAST qPCR master mix (Kapa biosystem) or HOT FIREPol EvaGreen qPCR Mix (Solis BioDyne). Relative abundance of mRNA was calculated by normalization to GAPDH mRNA. The reactions were carried out on a CFX96TM real-time system (BIO-RAD). For ChIP assay, DNA was extracted by phenol/chloroform/isoamyl alcohol, ethanol precipitated and re-suspended in water. In mRNA expression experiments, Ct values of every gene were normalized to the housekeeping GAPDH, while in ChIP assays, the normalization were calculated by the following formula: (X–IgG)/INPUT, where X is the Ct of the sequence of interest derived from the immunoprecipitated DNA bound to the protein of interest; IgG is the Ct of the same sequence derived from the DNA immunoprecipitated with an irrelevant antibody and INPUT is the Ct derived from the total DNA before immunoprecipitation. Primers for quantitative real time PCR (for ChIP assay) were obtained from MWG/Operon, with the following sequences: Forward: 5′ TTGGTGCTGGAAATACTGCG 3′, Reverse: 5′ CTCA GCGGGGAAGAGTTGTT 3′. Other primers used in this work are listed in Supplementary Table S2.

### Cell cycle and real-time cell proliferation assay

Cells were incubated in a 96-well plate (200 ul of medium/well) with the tested compounds for 24H and cell viability was quantitatively determined by a colorimetric MTT assay. In brief, MTT (5mg/ml) at 10% volume of culture media was added to each well and cells were further incubated for 2H at 37°C. Then supernatant containing MTT was replaced by 100μl of DMSO to dissolve formazan. Absorbance was then determined at 565nM by microplate reader. Cell survival was calculated and EC50 values were determined. Cell cycle was measured by FACS analysis at 24H after treatment using propidium iodide (PI) staining. Real time cell proliferation assay: PK9 were seeded 5000 cells/well in E-plates (Roche), in triplicates. The cell growth curves were automatically recorded on the xCELLigence System (Roche) in real time. The cell attachment was monitored every 15 minutes by a cell electronic system. The doubling times were calculated according with cell index. The cell index is an arbitrary unit for displaying inpedence. SCR or siRNAs transfection PK9 cells were performed by replacing cell medium, including untreated control to operate at the same working conditions.

### Two-dimensional electrophoresis

The experiment was performed according to previous protocols [52]. Proteins were extracted using a lysis buffer (8M urea, 4% CHAPS, 50mM dithioerythritol and 0,0002% Bromophenol blue) and rehydrated with 8M urea, 2% CHAPS, 20mM dithioerythritol, 0.8% IPG buffer, carrier ampholytes pH 6-11 linear. The first dimension isoelectric focusing (IEF) was performed in immobiline dry strips (GE) with a pH range from 7 to 4. IEF was performed on IPGphor (GE) according to the manufacturer recommendations. The gels were then equilibrated in 6M urea, 3% SDS, 375mM Tris pH 8.6, 30% glycerol, 2% DTE and then incubated with 3% iodoacetamide (IAA) and traces of bromophenol blue (BBP). The second dimension was performed using an 8% SDS-PAGE gel. Transfer and detection were carried out as previously described.

### Statistical analysis

The Data are presented as mean±SD and the standard deviation of the mean (SD) in this study were calculated for 3 replicates in each of the 3 independent experiments. Statistical comparisons were assessed with analysis of Student's test and One-way ANOVA calculated with GraphPad Prism version 5.0 for Windows (GraphPad Software). P<0.05 was considered statistically significant difference and P<0.01, and P<0.001 were considered as highly significant difference.

## SUPPLEMENTARY FIGURES AND TABLES







## References

[1] Sudol M (1994). Yes-associated protein (YAP65) is a proline-rich phosphoprotein that binds to the SH3 domain of the Yes proto-oncogene product. Oncogene.

[2] Zhao B, Wei X, Li W, Udan RS, Yang Q, Kim J, Xie J, Ikenoue T, Yu J, Li L, Zheng P, Ye K, Chinnaiyan A (2007). Inactivation of YAP oncoprotein by the Hippo pathway is involved in cell contact inhibition and tissue growth control. Genes Dev.

[3] Zhao B, Li L, Lu Q, Wang LH, Liu C-Y, Lei Q, Guan K-L (2011). Angiomotin is a novel Hippo pathway component that inhibits YAP oncoprotein. Genes Dev.

[4] Chan SW, Lim CJ, Chong YF, Pobbati A V, Huang C, Hong W (2011). Hippo pathway-independent restriction of TAZ and YAP by angiomotin. J Biol Chem.

[5] Azzolin L, Panciera T, Soligo S, Enzo E, Bicciato S, Dupont S, Bresolin S, Frasson C, Basso G, Guzzardo V, Fassina A, Cordenonsi M, Piccolo S (2014). YAP/TAZ incorporation in the β-catenin destruction complex orchestrates the Wnt response. Cell.

[6] Cai J, Maitra A, Anders RA, Taketo MM, Pan D (2015). β-Catenin destruction complex-independent regulation of Hippo-YAP signaling by APC in intestinal tumorigenesis. Genes Dev.

[7] Park J, Jeong S (2015). Wnt activated β-catenin and YAP proteins enhance the expression of non-coding RNA component of RNase MRP in colon cancer cells. Oncotarget.

[8] Zhao B, Ye X, Yu J, Li L, Li W, Li S, Yu J, Lin JD, Wang C-Y, Chinnaiyan AM, Lai Z-C, Guan K-L (2008). TEAD mediates YAP-dependent gene induction and growth control. Genes Dev.

[9] Fujii M, Toyoda T, Nakanishi H, Yatabe Y, Sato A, Matsudaira Y, Ito H, Murakami H, Kondo Y, Kondo E, Hida T, Tsujimura T, Osada H (2012). TGF-β synergizes with defects in the Hippo pathway to stimulate human malignant mesothelioma growth. J. Exp. Med.

[10] Strano S, Monti O, Pediconi N, Baccarini A, Fontemaggi G, Lapi E, Mantovani F, Damalas A, Citro G, Sacchi A, Del Sal G, Levrero M, Blandino G (2005). The transcriptional coactivator Yes-associated protein drives p73 gene-target specificity in response to DNA Damage. Mol Cell.

[11] Camargo FD, Gokhale S, Johnnidis JB, Fu D, Bell GW, Jaenisch R, Brummelkamp TR (2007). YAP1 increases organ size and expands undifferentiated progenitor cells. Curr Biol.

[12] Dong J, Feldmann G, Huang J, Wu S, Zhang N, Comerford SA, Gayyed MF, Anders RA, Maitra A, Pan D (2007). Elucidation of a universal size-control mechanism in Drosophila and mammals. Cell.

[13] Guo J, Kleeff J, Zhao Y, Li J, Giese T, Esposito I, Büchler MW, Korc M, Friess H (2006). Yes-associated protein (YAP65) in relation to Smad7 expression in human pancreatic ductal adenocarcinoma. Int J Mol Med.

[14] Diep CH, Zucker KM, Hostetter G, Watanabe A, Hu C, Munoz RM, Von Hoff DD, Han H (2012). Down-regulation of Yes Associated Protein 1 expression reduces cell proliferation and clonogenicity of pancreatic cancer cells. PLoS One.

[15] Zhang W, Nandakumar N, Shi Y, Manzano M, Smith A, Graham G, Gupta S, Vietsch EE, Laughlin SZ, Wadhwa M, Chetram M, Joshi M, Wang F (2014). Downstream of mutant KRAS, the transcription regulator YAP is essential for neoplastic progression to pancreatic ductal adenocarcinoma. Sci Signal.

[16] Shao DD, Xue W, Krall EB, Bhutkar A, Piccioni F, Wang X, Schinzel AC, Sood S, Rosenbluh J, Kim JW, Zwang Y, Roberts TM, Root DE (2014). KRAS and YAP1 converge to regulate EMT and tumor survival. Cell.

[17] Jones S, Zhang X, Parsons DW, Lin JC-H, Leary RJ, Angenendt P, Mankoo P, Carter H, Kamiyama H, Jimeno A, Hong SM, Fu B, Lin MT (2008). Core signaling pathways in human pancreatic cancers revealed by global genomic analyses. Science.

[18] Coulouarn C, Factor VM, Thorgeirsson SS (2008). Transforming growth factor-beta gene expression signature in mouse hepatocytes predicts clinical outcome in human cancer. Hepatology.

[19] Neuzillet C, de Gramont A, Tijeras-Raballand A, de Mestier L, Cros J, Faivre S, Raymond E (2014). Perspectives of TGF-β inhibition in pancreatic and hepatocellular carcinomas. Oncotarget.

[20] Grosse-Gehling P, Fargeas CA, Dittfeld C, Garbe Y, Alison MR, Corbeil D, Kunz-Schughart LA (2013). CD133 as a biomarker for putative cancer stem cells in solid tumours: limitations, problems and challenges. J Pathol.

[21] Nomura A, Banerjee S, Chugh R, Dudeja V, Yamamoto M, Vickers SM, Saluja AK (2015). CD133 initiates tumors, induces epithelial-mesenchymal transition and increases metastasis in pancreatic cancer. Oncotarget.

[22] Nagathihalli NS, Merchant NB (2012). Src-mediated regulation of E-cadherin and EMT in pancreatic cancer. Front Biosci.

[23] Ding Q, Miyazaki Y, Tsukasa K, Matsubara S, Yoshimitsu M, Takao S (2014). CD133 facilitates epithelial-mesenchymal transition through interaction with the ERK pathway in pancreatic cancer metastasis. Mol Cancer.

[24] Kim N, Koh E, Chen X, Gumbiner BM (2011). E-cadherin mediates contact inhibition of proliferation through Hippo signaling-pathway components. Proc Natl Acad Sci U S A.

[25] Shen Z, Stanger BZ (2015). YAP regulates S-phase entry in endothelial cells. PLoS One.

[26] Levitzki A, Mishani E (2006). Tyrphostins and other tyrosine kinase inhibitors. Annu Rev Biochem.

[27] Almoguera C, Shibata D, Forrester K, Martin J, Arnheim N, Perucho M (1988). Most human carcinomas of the exocrine pancreas contain mutant c-K-ras genes. Cell.

[28] Hers I, Tavaré JM, Denton RM (1999). The protein kinase C inhibitors bisindolylmaleimide I (GF 109203x) and IX (Ro 31-8220) are potent inhibitors of glycogen synthase kinase-3 activity. FEBS Lett.

[29] Kinehara M, Kawamura S, Tateyama D, Suga M, Matsumura H, Mimura S, Hirayama N, Hirata M, Uchio-Yamada K, Kohara A, Yanagihara K, Furue MK (2013). Protein kinase C regulates human pluripotent stem cell self-renewal. PLoS One.

[30] Brehmer D, Godl K, Zech B, Wissing J, Daub H (2004). Proteome-wide identification of cellular targets affected by bisindolylmaleimide-type protein kinase C inhibitors. Mol Cell Proteomics.

[31] Zhang H, Liu C-Y, Zha Z-Y, Zhao B, Yao J, Zhao S, Xiong Y, Lei Q-Y, Guan K-L (2009). TEAD transcription factors mediate the function of TAZ in cell growth and epithelial-mesenchymal transition. J Biol Chem.

[32] Deng Y-Z, Chen P-P, Wang Y, Yin D, Koeffler HP, Li B, Tong X-J, Xie D (2007). Connective tissue growth factor is overexpressed in esophageal squamous cell carcinoma and promotes tumorigenicity through beta-catenin-T-cell factor/Lef signaling. J Biol Chem.

[33] Overholtzer M, Zhang J, Smolen G a, Muir B, Li W, Sgroi DC, Deng C-X, Brugge JS, Haber DA (2006). Transforming properties of YAP, a candidate oncogene on the chromosome 11q22 amplicon. Proc Natl Acad Sci U S A.

[34] Hall CA, Wang R, Miao J, Oliva E, Shen X, Wheeler T, Hilsenbeck SG, Orsulic S, Goode S (2010). Hippo pathway effector Yap is an ovarian cancer oncogene. Cancer Res.

[35] Reddy BVVG, Irvine KD (2013). Regulation of Hippo signaling by EGFR-MAPK signaling through Ajuba family proteins. Dev Cell.

[36] Urtasun R, Latasa MU, Demartis MI, Balzani S, Goñi S, Garcia-Irigoyen O, Elizalde M, Azcona M, Pascale RM, Feo F, Bioulac-Sage P, Balabaud C, Muntané J (2011). Connective tissue growth factor autocriny in human hepatocellular carcinoma: oncogenic role and regulation by epidermal growth factor receptor/yes-associated protein-mediated activation. Hepatology.

[37] You B, Yang Y-L, Xu Z, Dai Y, Liu S, Mao J-H, Tetsu O, Li H, Jablons DM, You L (2015). Inhibition of ERK1/2 down-regulates the Hippo/YAP signaling pathway in human NSCLC cells. Oncotarget.

[38] Haskins JW, Nguyen DX, Stern DF (2014). Neuregulin 1-activated ERBB4 interacts with YAP to induce Hippo pathway target genes and promote cell migration. Sci Signal.

[39] Wang JP, Wu C-Y, Yeh Y-C, Shyr Y-M, Wu Y-Y, Kuo C-Y, Hung Y-P, Chen M-H, Lee W-P, Luo J-C, Chao Y, Li C-P (2015). Erlotinib is effective in pancreatic cancer with epidermal growth factor receptor mutations: a randomized, open-label, prospective trial. Oncotarget.

[40] Orford K, Crockett C, Jensen JP, Weissman AM, Byers SW (1997). Serine phosphorylation-regulated ubiquitination and degradation of beta-catenin. J Biol Chem.

[41] Easwaran V, Song V, Polakis P, Byers S (1999). The ubiquitin-proteasome pathway and serine kinase activity modulate adenomatous polyposis coli protein-mediated regulation of beta-catenin-lymphocyte enhancer-binding factor signaling. J Biol Chem.

[42] Cho M, Park S, Gwak J, Kim D-E, Yea SS, Shin J-G, Oh S (2008). Bisindoylmaleimide I suppresses adipocyte differentiation through stabilization of intracellular beta-catenin protein. Biochem Biophys Res Commun.

[43] Zhou F, Huang H, Zhang L (2012). Bisindoylmaleimide I enhances osteogenic differentiation. Protein Cell.

[44] Chen Y, Blom IE, Sa S, Goldschmeding R, Abraham DJ, Leask A (2002). CTGF expression in mesangial cells: involvement of SMADs, MAP kinase, and PKC. Kidney Int.

[45] Bahammam M, Black SA, Sume SS, Assaggaf MA, Faibish M, Trackman PC (2013). Requirement for active glycogen synthase kinase-3β in TGF-β1 upregulation of connective tissue growth factor (CCN2/CTGF) levels in human gingival fibroblasts. Am J Physiol Cell Physiol.

[46] Lee SJ, Kang JH, Choi SY, Kwon OS (2013). PKCδ as a regulator for TGF-β-stimulated connective tissue growth factor production in human hepatocarcinoma (HepG2) cells. Biochem J.

[47] Jones S, Zhang X, Parsons DW, Lin JC-H, Leary RJ, Angenendt P, Mankoo P, Carter H, Kamiyama H, Jimeno A, Hong S-M, Fu B, Lin M-T (2008). Core signaling pathways in human pancreatic cancers revealed by global genomic analyses. Science.

[48] Zhang H, von Gise A, Liu Q, Hu T, Tian X, He L, Pu W, Huang X, He L, Cai C-L, Camargo FD, Pu WT, Zhou B (2014). Yap1 is required for endothelial to mesenchymal transition of the atrioventricular cushion. J Biol Chem.

[49] Taccioli C, Sorrentino G, Zannini A, Caroli J, Beneventano D, Anderlucci L, Lolli M, Bicciato S, Del Sal G (2015). MDP, a database linking drug response data to genomic information, identifies dasatinib and statins as a combinatorial strategy to inhibit YAP/TAZ in cancer cells. Oncotarget.

[50] Bhukhai K, Suksen K, Bhummaphan N, Janjorn K, Thongon N, Tantikanlayaporn D, Piyachaturawat P, Suksamrarn A, Chairoungdua A (2012). A phytoestrogen diarylheptanoid mediates estrogen receptor/Akt/glycogen synthase kinase 3β protein-dependent activation of the Wnt/β-catenin signaling pathway. J Biol Chem.

[51] D'Agostino VG, Adami V, Provenzani A (2013). A Novel High Throughput Biochemical Assay to Evaluate the HuR Protein-RNA Complex Formation. PLoS One.

[52] Latorre E, Tebaldi T, Viero G, Spartà AM, Quattrone A, Provenzani A (2012). Downregulation of HuR as a new mechanism of doxorubicin resistance in breast cancer cells. Mol Cancer.

