# Supplementary material for: The GSK3β inhibitor BIS I reverts YAP-dependent EMT signature in PDAC cell lines by decreasing SMADs expression level
Source: Oncotarget. 2016 Mar 28;7(18):26551–66. doi: 10.18632/oncotarget.8437 (PMC5041998; doi:10.18632/oncotarget.8437)
Supplement: Supplementary file 1 [file oncotarget-07-26551-s001.pdf]

SUPPLEMENTARY FIGURES AND TABLES

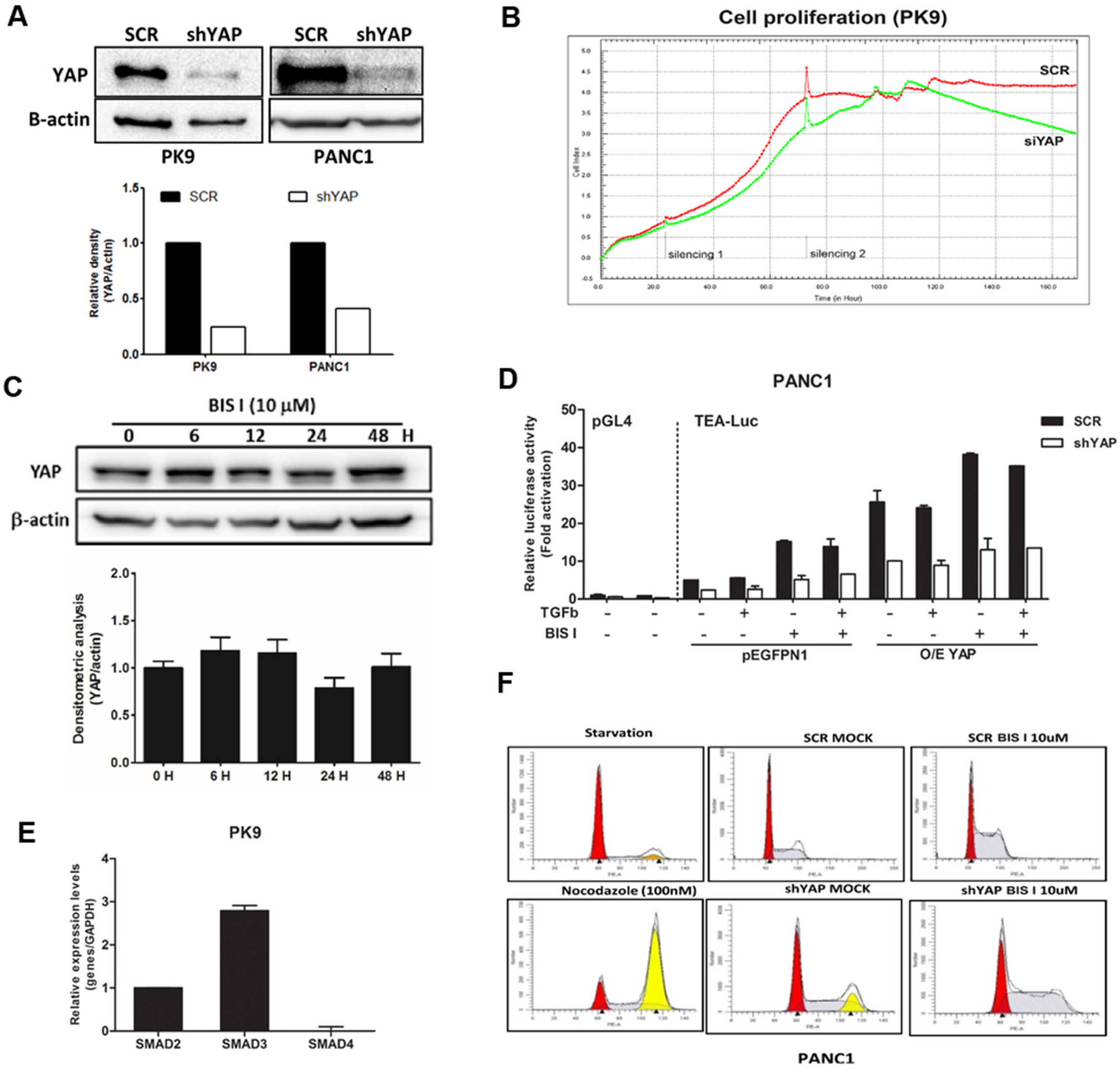

Supplementary Figure S1: A. Silencing efficiency using transduction of short-hairpin RNAs against YAP in PDAC cell lines. Representative western blots of YAP levels and β-catenin in stably silenced PDAC cell lines. Densitometric analysis was performed using Image J software (below panel). **B. Genetic ablation of YAP slows cell proliferation rate.** PK9 were seeded 5000 cells/well in E-plates (Roche), in triplicates. Cell indexes of growth were automatically recorded by the xCELLigence System (Roche) in real time. **C. Endogenous protein expression of YAP does not change during BIS I treatment.** Representative western blots showing the expression level of YAP during a time course treatment of BIS I 10μM. Relative protein expression was quantified. **D. TGF-β independent regulation of TEA reporter activity.** SCR or stably YAP-silenced PANC1 cells were transiently co-transfected with pEGFPN1 or YAP (O/E YAP) and TEA reporter (8xGTIIC-Luc reporter). They were treated with BIS I 5μM in the presence and absence of TGF-β for 24H. The firefly luciferase signals were normalized to the ones of *Renilla.* (mean±SD from biological triplicates). **E. SMADs gene expression level in PK9.** Expression level of *SMAD2, SMAD3* and *SMAD3* were measured by qRT-PCR. **F. BIS I treatment and genetic ablation of YAP induce accumulation of S-phase cell sub-populations.** PANC1 cells were treated with BIS I 10μM for 24H. Starvation and Nocodazole conditions were used as controls for G0/G1 and S-phase accumulation, respectively. Cell cycle phase analysis was done using ModFit LT 3.2 software and the Sync Wizard model.

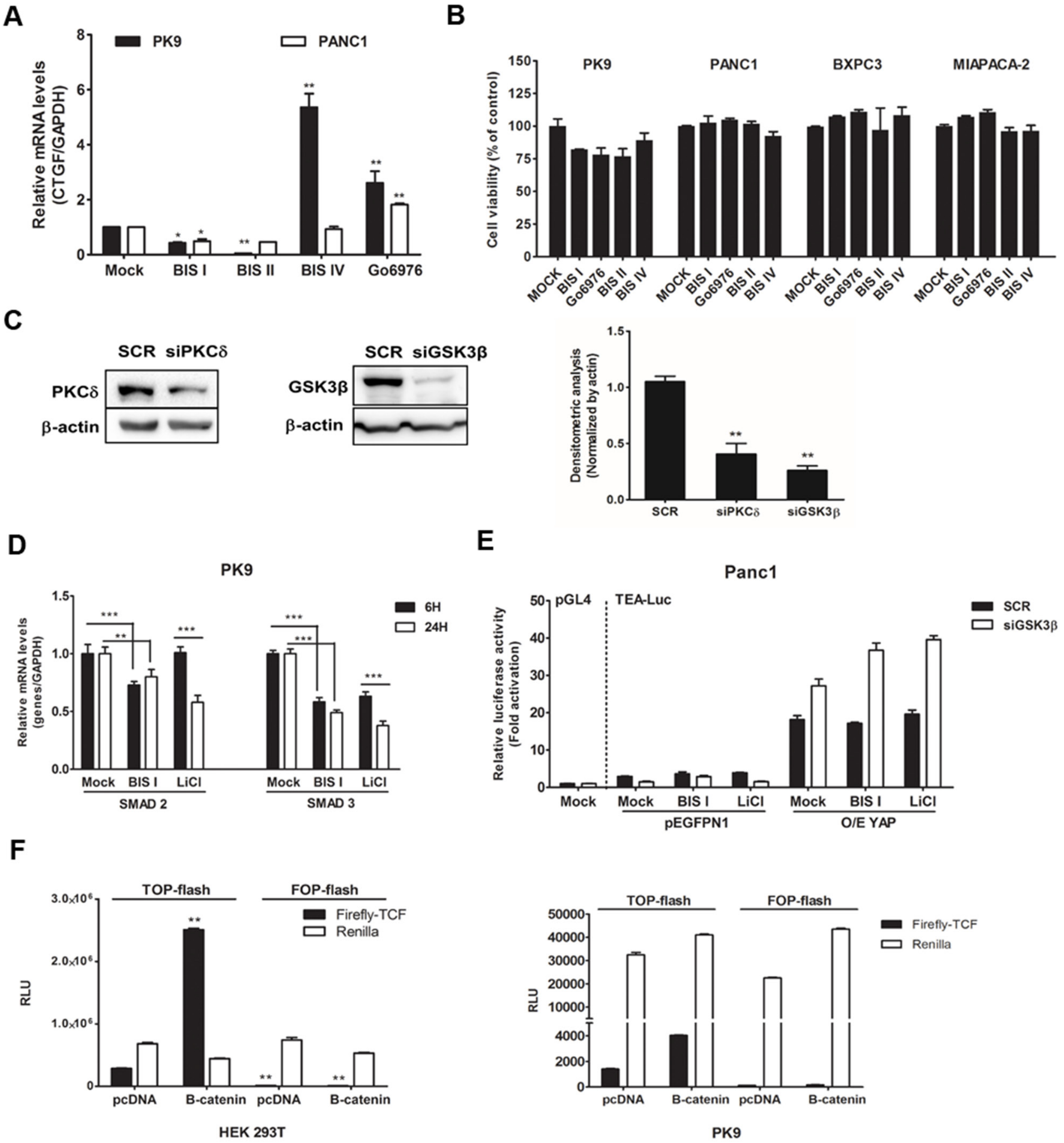

Supplementary Figure S2: A. Opposite effects of bisindolylmaleimide kinase inhibitors on CTGF mRNA expression. PK9 and PANC1 cells were treated with DMSO, BIS I, Go6976, BIS II, and BIS IV compounds (5µM) for 24H and qRT-PCRs were performed to quantify *CTGF* expression relative to *GAPDH*. (*p<0.05 and **p<0.01 versus MOCK). **B. PDAC cell lines show different sensitivity upon treatment with Bisindolylmaleimide family of compounds.** Cell viability of PDAC cell lines treated with as in (A) for 24H and evaluated by MTT assay. **C. Silencing of PKCδ or GSK3β in PK9,** PK9 cells were incubated with siRNA targeting PKCδ, GSK3β, and non-targeting control (SCR) for 72H. Transient knock down of PKCδ and GSK3β were confirmed by immunoblotting against PKCδ and GSK3β antibodies. The relative intensities of the bands normalized by β-actin is indicated. (**p<0.01 versus SCR). **D. Loss of SMADs mRNAs during prolonged treatment with LiCl.** PK9 cells were treated with BIS I 10µM or LiCl 50mM for 24H. Quantitative RT-PCRs of *SMAD2* and *SMAD3* relative to *GAPDH* expression with respect to MOCK are presented as mean±SD. (***p<0.01 versus MOCK). **E. Genetic ablation of GSK3β activates YAP/TEAD reporter activity.** PANC1 cells were incubated with siRNA targeting GSK3β, and non-targeting control (SCR) for 48H and then they were transiently co-transfected with pEGFPN1 or YAP (O/E YAP) and TEA reporter. Twenty-four hours after transfection, cells were then treated with BIS I 5µM and LiCl 50mM for 24H. Right panel: YAP is required for TEA reporter activation. The firefly luciferase signals were normalized to the ones of *Renilla.* **F. β-catenin reporter activity in HEK293T and PK9 cells.** HEK293T cells (Left) and PK9 cells (right) were transiently transfected with Wild-type TCF binding site (TOP-flash) which is responsive to β-catenin expression or with mutated binding site (FOP-flash). The firefly luciferase signals were normalized to the ones of *Renilla.*

Supplementary Table S1: List of hits derived from High-Content Screening of the kinase inhibitor library.

See Supplementary File 1

Supplementary Table S2: List of primers used for qRT-PCRs.

See Supplementary File 2
